# Ecological Psychology and Enactivism: Perceptually-Guided Action vs. Sensation-Based Enaction^1^

**DOI:** 10.3389/fpsyg.2020.01270

**Published:** 2020-07-14

**Authors:** Catherine Read, Agnes Szokolszky

**Affiliations:** ^1^Plant Biology, Rutgers University, New Jersey, NJ, United States; ^2^Department of Psychology, Ithaca College, New York, NY, United States; ^3^Department of Cognitive and Neuropsychology, Institute of Psychology, Szeged University, University of Szeged, Szeged, Hungary

**Keywords:** Ecological Psychology, Enactivism, direct perception, sensation based perception theories, retinal image theory, ecological mutualism, James J. Gibson, Francesco Varela

## Abstract

Ecological Psychology and Enactivism both challenge representationist cognitive science, but the two approaches have only begun to engage in dialogue. Further conceptual clarification is required in which differences are as important as common ground. This paper enters the dialogue by focusing on important differences. After a brief account of the parallel histories of Ecological Psychology and Enactivism, we cover incompatibility between them regarding their theories of sensation and perception. First, we show how and why in ecological theory perception is, crutially, not based on sensation. We elucidate this idea by examining the biological roots of work in the two fields, concentrating on Gibson and Varela and Maturana. We expound an ecological critique of any sensation based approach to perception by detailing two topics: classic retinal image theories and perception in single-celled organisms. The second main point emphasizes the importance of the idea of organism-environment mutuality and its difference from structural coupling of sensations and motor behavior. We point out how ecological—phenomenological methods of inquiry grow out of mutualism and compare Gibson's idea of visual kinesthesis to Merleau-Ponty's idea of the lived body. Third, we conclude that Ecological Psychology and varieties of Enactivism are laying down different paths to pursue related goals. Thus, convergence of Ecological Psychology and Enactivism is not possible given their conflicting assumptions, but cross-fertilization is possible and desirable.

The seemingly paradoxical assertion will be made that perception is not based on sensation. That is, it is not based on having sensations…but it is surely based on detecting information.

James (Gibson, 1966)

..partly because of the difficulty of Merleau's rhetoric…, and partly because he needed a theory of perception that didn't then exist and now does, I have concentrated rather on trying to understand and expound Gibson than on trying to bring the American and the French thinker together.

Marjorie (Grene, 1995)

## Introduction

After a brief account of the parallel histories of Ecological Psychology and Enactivism, we cover three main points about the relations between them. First, perception is distinct from sensation.

This idea is elaborated by examining the biological roots of work in the two fields, concentrating on Gibson in Ecological Psychology and Varela and Maturana in Enactivism. We cover critical assumptions about perception and about methods of study. The second main point emphasizes the importance of the idea of organism-environment mutuality, and the differences between the ecological idea of mutuality and the enactivist idea of autopoesis or self-creation. Third, we conclude by contrasting the two approaches, pointing out areas that each has yet to develop. We conclude that a convergence of Ecological Psychology and Enactivism is not possible given their conflicting assumptions, but that cross-fertilization is possible and desirable.

Ecological Psychology is the oldest radical challenger of classical representationist cognitive science with its seven-decade-long history. The germinal books for this approach are James J. Gibson's *The Senses Considered as Perceptual Systems* (1966) and *The Ecological Approach To Visual Perception* (1979, 2015). During these decades (between the 1970's and the 1990's) ecological psychologists kept elaborating, theoretically and empirically, its core idea: that perceiving—acting organisms are in direct epistemic contact with their environment via ecological specification and affordances. In the meantime, Ecological Psychology has branched out from its core experimental studies on action and perception into diverse contexts including human development, movement science, social dynamics, ecological robotics, and other cognitive topics such as language and metaphor use.

The enactive approach is based on work by Maturana and Varela (1988), and was succintly articulated for the wider audience in the early 1990's, in the germinal book *The embodied mind: Cognitive science and human experience* by Varela et al. (1993). In this book Varela et al. launched their own challenge to the cognitivist paradigm, with the core idea that cognition is an embodied, lived process, based on self-organizing and recurrent sensorimotor patterns. Enactivism quickly became influential, and gradually three main versions appeared: autopoetic, sensori-motor, and radical enactivism (cf. Ward et al., 2017). However, they all are conceptual descendants of the enactivist program, as defined by Varela et al. (1993).

In *The embodied mind* Varela et al. acknowledged, but also firmly criticized, Ecological Psychology claiming that Gibson understood environmental structures as objective, pre-specified properties to which the organism must respond (Varela et al., 1993, pp. 203–204). Clearly, this was a one-sided and largely inaccurate reading of Gibson's theory, as pointed out later by others (e.g., Fultot et al., 2016), and it disallowed taking the Ecological program as a partner to engage with. Not until recently did representatives of Ecological Psychology react to this criticism and engage in discussion with Enactivism. Perhaps a “a full-blown” post-cognitivist science of the mind might need both ecological and enactivist insights (Heft, 2001; Chemero, 2009; Rietveld and Kiverstein, 2014; van Dijk et al., 2015; McGann, 2016; Hutto and Myin, 2017; Bruineberg et al., 2019).

An important step was a dialogue in the 2016 Special Issue of Constructivist Foundations. The target article by Fultot et al. pointed out the misreadings of Ecological Psychology by Varela et al. and critiqued Enactivism on the basis of being, in the final analysis, internalist and concluded that enacting a world seems to be “constructivism in the most traditional sense” (par.50). Now it was on the enactivist side to claim this as an “uncheritable” reading (Stapleton, 2016).

Difficulties for clarification abound as neither Ecological Psychology nor Enactivism comprises a fully developed, homogeneous set of views. As apparent in recollections by ecological scientists^2^, in the early decades of Ecological Psychology cognitive science presented a hostile cognitivist environment. Under these circumstances Ecological Psychology focused “inwards” and worked on turning basic insights by James J. Gibson into a full blown theory of action and perception and workable empirical research programme.

In the exchanges between Ecological Psychology and Enactivism some significant questions and critiques have surfaced on both sides. In a simplified summary, the major questions about Ecological Psychology on the enactivist side are the following (based on the target article and the commentaries in 2016 Special Issue of Constructivist Foundations): (1) Ecological Psychology puts too much load and emphasis on symmetry principles in organism—environment mutuality; (2) Thereby Ecological Psychology does not do justice to the autonomy, subjectivity, perceptual consciousness, historicity of the agent side, which is necessary to account for an active agent with a self; (3) In Ecological Psychology descriptions of the environment (affordances, specification) refer to pre-existing structures that are not dependant on experience and, therefore, are not truly relational; (4) Ecological Psychology ignores the subpersonal level of emergent processes, that is, the level of the physical basis of perceptual experience (on the distinction between personal and subpersonal see Thompson and Cosmelli, 2011; Roberts, 2018). The major questions on the ecological side are the following: (1) Enactivism embraces subjectivity and constructive processes and thereby opens up the door to dualism; (2) Enactivism takes sensorimotor functioning as the starting point which retains the idea of the “poverty of the stimulus” and does not explain how meaning emerges from something non-meaningful; (3) Thereby Enactivism also fails to establish how the organism/agent is in direct epistemic contact with its environment; (4) Enactivism does not treat the organismic level as a distinguished level of analysis, and, thereby, does not satisfy truly ecological mutualism.

Issues and questions exist, however, not just concerning the “other side,” but also about “own sides.” Fundamental questions are still being tackled within each framework. Versions of Enactivism lay out different ideas about agency, embodiment, or sensorimotor contingencies (cf. Ward et al., 2017). Whereas within the framework of Ecological Psychology serious discussion continues regarding the interpretation of such fundamental concepts as the environment, information, and affordances (see e.g., Chemero, 2009; Read and Szokolszky, 2018; van Dijk and Myin, 2019).

We take the above questions as opportunities for reflection. Earlier we briefly discussed the Enactivism-Ecological Psychology relationship (cf. Szokolszky et al., 2019). We highlighted basic strategic and conceptual differences in their ways of explaining animal—environment mutuality; however, we concluded that a dialogue benefits both parties. This time we continue the dialogue by focusing on some of the ecological questions about Enactivism, but also on some critical questions related to Ecological Psychology.

In this paper we focus on two main points of difference between Ecological Psychology and Enactivism. Our first main point is to emphasize that perception is not sensation-based. We will elaborate the argument against sensorimotor capacities and contingencies as the foundation for a psychology of perceiving and knowing. Along with others committed to Ecological Psychology we claim that this is a fundamental point of difference that needs to be squarely addressed (cf. Szokolszky et al., 2019). We agree with Michaels and Palatinus (2014, p.19) that Ecological Psychology comes “as a package deal”: the major principles of Ecological Psychology are “deeply connected and intertwined. To subscribe to some and discard the others always entails contradiction.” We elaborate this distinction between perception and sensations by examining the biological roots of work in the two fields, concentrating on Gibson in Ecological Psychology and Varela and Maturana in Enactivism. Here we go over two prototypical topics: the retinal image theory of vision, and perception by single-celled organisms. We cover critical assumptions about perception and about methods of study.

The second main point emphasizes the importance of the idea of organism-environment mutuality and how it is more than organism-environment interaction or coupling. Ecological Psychology has developed the concept of mutuality and Enactivism has focused on individuals (usually conscious human beings) interacting with the world. These two concepts are radically different and should not be confused.

In the final analysis, we conclude that the concepts of sensorimotor action, -agent, and -life are profoundly different from the concepts of ecological action, -agent and -life. These profound differences must be recognized and acknowledged. This does not mean, however, that there is no point to the dialogue. Both Enactivism and Ecological Psychology are developing enterprises that still need to give elaborate answers to questions regarding brain level processes, knowing and feeling, consciousness, and phenomenological experience. Ecological Psychology has a coherent account of contact with the environment, however, the role of experience in perceiving/acting, or even the importance of experience, has not been developed in ecological work to date (see related points in Kadar and Effken, 1994, 2006). This even though Gibson drew on experiences (that anyone with a functioning perceptual system can share) to develop his theory of how the structure of the surround is detected^3^. Enactionism draws heavily on phenomenology, but its assumptions on the sensorimotor foundations of agency are not compatible with Ecological Psychology. The paper concludes that the goal is not to seek convergence, but to keep attending to each other and leaving open the possibility for potential cross-fertilization.

## First Main Point: Perception Is Not Based on Sensation

A fundamental difference exists between Ecological Psychology and Enactivism regarding the interpretation of the role of sensation and perception as the foundation for action. Although they both share an emphasis on action, Enactivism takes sensorimotor capacities and contingencies as the basis for action, whereas for Ecological Psychology perception is not constituted by sensation and, therefore, perceiving/acting based on affordances of the surround is the cornerstone of cognitive functioning. Therein lie deep differences in the concept of “enaction” and ecological perception/action.

Varela et al. (1993) claim (p. 173) that enaction means that: (1) perception consist of perceptually-guided action; and that (2) cognitive structures emerge from the recurrent sensorimotor patterns that enable action to be perceptually guided^4^. On the other hand, Gibson claims that having sensations is not perception, rather, perception is the functioning of perceptual systems that include the whole organism. Clearly, these are two thoroughly opposing views. At stake is the very foundation on which a viable alternative to representationism can be built. Why and how the two approaches come to build on these different foundations and what are the consequences? This is a complex question that needs attention.

Both approaches have roots in biology, but their starting points and paths of development are diametrically opposite. We are looking for answers by exploring the biological roots of enactive and ecological explanations.

### The Biological Roots of Direct Perception Ecological Explanations and Sensation Based Enactive Explanations

The roots of the ecological approach to perception (Gibson) and the embodied approach to the mind (Varela and Maturana) extend into biology, but in very distinct ways. In the service of understanding the contrasts and relations of these two approaches we now briefly compare the biological basis and assumptions of the two theories by considering early work in biology by Varela (1997) on patterns of life and by Maturana et al. (1960), Maturana et al. (1960) on vision in the frog. In contrast, we review work by Gibson (1979) countering the retinal image theory of perception and by Pittenger and Dent (1988) on a direct perception account of bacterial chemotaxis.

Varela equates living organisms with living systems and, consequently, gives organisms the qualities of what he defines as natural systems. Organisms are a process of constituting an identity and identity refers to coherence; identity is not a structure, but a process, and it is not mentalistic or personal (Varela, 1997). In describing autopoiesis, Varela takes the example of the bacterial cell which has the capacity to produce all the components that comprise a distinct, bounded unit. How a natural system differentiates is not considered, nor is reproduction. The latter is seen as a process that is an “added complexity superimposed on a more basic entity” (p. 75).

Note the contrast to Gibson's (1979) focus on what might be called “conditions of life.” Gibson begins with a perceiving organism, not a natural system, and especially not one that is self-made. If one starts from the point of view of a system, and the formal logic of systems, then the organism tends to become “just” a system, or an aspect of a system. If one starts with the organism, then how it moves, lives, reproduces, and dies in its conditions/surrounds leads to different research questions (e.g., Sheldrake, 1981; Gilbert and Sarkar, 2000). The organicist approach leads to questions of mutualism, adaptation, and affordance, however those are defined. And it leads to very different questions and assumptions about perception.

Maturana, who researched the frog visual system, is an example of a theorist who accepts and assumes the mentalist account of perception (e.g., Lettvin et al., 1959; Maturana et al., 1960). Some of the assumptions about perception that the researchers hold are evident from their description of the process of frogs seeing and catching prey. For example, frog vision is described as using visual clues, that the objects toward which the frogs act have certain features, such as movement, size, contrast, color. The analogy to human reading is used to characterize frog recognition of appropriate prey:

“Just as we are able to read and to recognize shapes under the most varied conditions, so are frogs able to see their prey and to feed upon it under the bright light of midday or under the twilight of morning or evening, whether this be in their natural environment or in a small cage in the laboratory” (Maturana et al., 1960, p. 129).

They ask how a frog recognizes prey or enemy and assert that:

“To survive, a frog needs to react rapidly, either to catch a prey or to escape an enemy. To do this, the pattern of light and dark that is the original image formed on the retina has to be analyzed, sooner or later, to select it the features which define the universals” (Maturana et al., 1960, p. 1).

This constitutes a straightforward and clear statement of the retinal image theory of vision, a theory that is assumed to pertain to any organism with (chambered) eyes. How does this theory with its set of assumptions about what constitutes perception coordinate with the type of studies conducted and the conclusions drawn from the studies? The authors argue that if the retina “performed the analysis” behavior could be quicker and more adaptive. “Thus, for anatomical reasons, the retina should be expected to perform the first step in the analysis of the visual image and to transmit the abstracted information to the visual centers” (p. 130). Therefore, the retinal cells are “mapped” for their patterns of activation in terms of a binary “on/off” logic. One conclusion from the detailed and complex results of the anatomical studies is that the retina is the first point of “transformation” by summing, and so forth, of the image. (Perhaps this move is way of doing away with the problematic “image” as soon as possible?).

Does this approach to perception persist in later work on embodied cognition (e.g., Maturana and Varela, 1980, 1988; Varela et al., 1993)? Maturana and Varela (1980, 1988) analyze living systems, as opposed to organisms, let alone animate organisms that perceive and act. Living systems are defined as units of interactions that exist in an ambience. Living systems cannot be understood independently from that part of the ambience with which they interact (the niche) nor can that part of the ambience be understood independently of the living system that defines it. The living system is seen as hierarchical, that is, the first order system is made up of cells, the second order of organisms, and the third order of organizations of organisms. “Reproduction and evolution are not essential for the living organization” (1988, p. 11). Cognition is defined as the acting or behaving in the domain of interactions in which a system can act to maintain itself. Living is a process of cognition, whether the system includes a nervous system or not. Living systems have internal states that can be changed relative to the maintenance of the system, that is, to maintenance of its identity. Sensors of an animal are modified by physical events, but a nervous system allows the internal states to be modified by “pure relations” viz.,

“The nervous system expands the cognitive domain of the living system by making possible interactions with ‘pure relations'; it does not create cognition.” (Maturana and Varela, 1988, p. 13).

On this account perception is defined as based on physical changes in sensors (the subpersonal) that then are somehow acted upon as internal states. In more general, i.e., less technical terms, living beings are continually self-producing and cell metabolism is the clearest example of this phenomenon (Maturana and Varela, 1988). Consequently, perception is defined as sensation-based, for example, in the case of a frog seeing and targeting a fly as prey. On this view there is an internal correlation between “the place where the retina receives a given perturbation and the muscular contractions that move the tongue, the mouth, the neck, and, in fact, the frog's entire body.” (p. 126). This “correlation” is termed “sensorimotor coordination.” As applied to single-celled organisms, e.g., amoebae, this idea leads to descriptions of the formation of a pseudopod and the consequent movement of the cell toward a protozoan in terms of chemical changes at the surface of the membrane of the cell and the maintenance of an “*internal correlation* between the degree of change of its membrane and those protoplasmic changes we see as pseudopods.” (italics in the original) (p. 147). On this view, the amoeba when engulfing and digesting a protozoan is an example of what Varela (1997) described as the simplest living system (see above). It is safe to say that this early sensation-based approach by Maturana persists in later works.

A next step, chronologically, was to take the phenomenological definition of perception as *reflection upon experience*, and to follow Merleau-Ponty in taking this reflection to *create* the relation between self and world (Varela et al., 1993). Thus, experience of one's own body in the world becomes the basis of “embodied action.” For enactivists, sensory and motor processes are equated with and taken as defining perception and action. And it is claimed that perception consists in perceptually guided action and that cognitive structures emerge from the recurrent sensorimotor patterns that allow perception to guide action (Varela et al., 1993, p. 173) (cf. frog vision and prey capture). Therefore, “the reference point for understanding perception is no longer a pregiven, perceiver independent world but rather the sensorimotor structure of the perceiver (the way in which the nervous system links sensory and motor surfaces)” (Varela et al., 1993, p. 173).

We are using these quotations in order to be able to point out the contrasts—and later also possible connections—with the ecological approach to perception. Next, however, we focus on the ecological critique of sensation-based functioning—a cardinal point for James Gibson and subsequent researchers.

### The Ecological Critique of Any Sensation Based Approach to Perception

Does sensorimotor coordination equal perceptually-guided action? The answer from Ecological Psychology is “no.” In both the 1966 book on The Senses Considered as Perceptual Systems and the 1979 book on The Ecological Approach to Visual Perception, Gibson endeavored to lay out the differences between the senses as physiological processes and perception as the active resonance to an actual surround. We will use his critique of the retinal image theory of vision as a example of the contrast between functioning of the senses and what he called direct perception. First we examine retinal image theories briefly.

### Classic Retinal Image Theories and Gibson's Rejection

**Figure 1:**
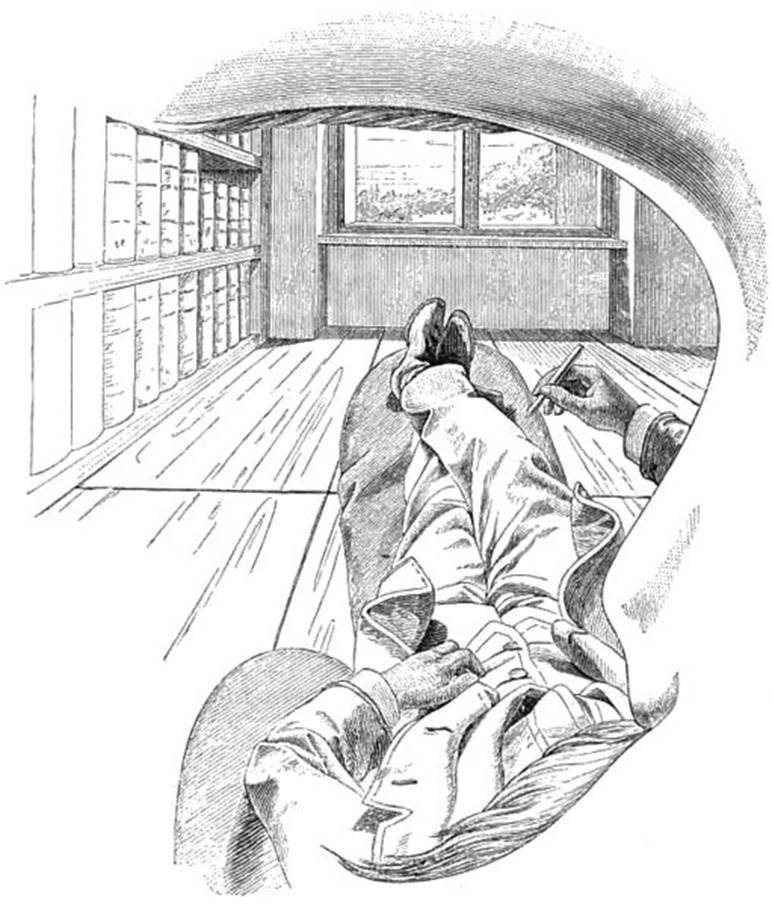
Ernst Mach Self Portrait (public domain). The assertion then is correct that the world consists only of our sensations (Gibson, 1979, p. 10).

The retinal image theory is a specific case of a general set of assumptions about the perception of the world that limit perception to operations on the “products” of the senses, and therefore, assume that only sensations (with cognitive transformations) are perceived. We briefly refer to George Berkeley and Ernst Mach as classic formulators of the retinal image theory of vision. In the retinal image tradition Berkeley (1709) assumes that objects in the environment are taken by perceivers to be of determinate size and place, even though the “visual appearance” continually changes, here the visual appearance means a visual image that is assumed to be cast on the retina (cf. Maturana, see above). Mach, in the Analysis of Sensations (1897), stated:

“Colors, sounds, temperatures, pressures, spaces, times, and so forth, are connected with one another in manifold ways; and with them are associated dispositions of mind, feelings, and volitions. Out of this fabric, that which is relatively more fixed and permanent stands prominently forth, engraves itself on the memory, and expresses itself in language.” (Mach, 1897, p. 1) See Figure 1 above.

**Figure 2:**
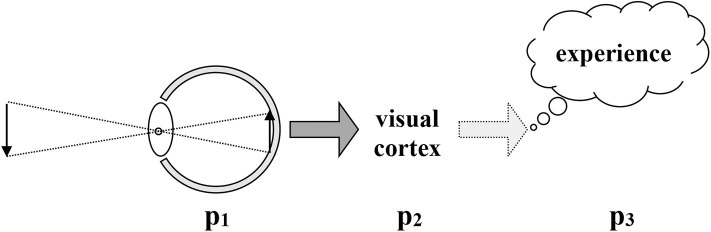
Traditional causal chain in vision Drawn by Robert Shaw, used with permission.

Modern assumptions about vision, in both biology and psychology, are captured in Figure 2 below. The basic account is that there is a causal line from light rays and lenses to points on a tissue of retinal cells, to firing patterns in nerve and brain, to experience. Enactivist approaches critique the idea of a linear causal chain, and instead use the idea of embedded levels of circular self-sustaining activity. Although that move does away with mental processing as a step somehow separate from brain activity and before perceiving, it accepts, however, the basic assumptions of retinal image theories, as we have seen above. Therefore, we are justified in laying out the deeper critique of retinal images that J.J. Gibson presented.

Gibson claims that the puzzle of rearranging, or correcting, or interpreting the messages of the retinal points is insoluble^5^. He offered ecological optics to provide a new starting point for a theory of perception based not on form-sensations (or perceptions) from the retina, but on information pickup from the ambient light (Gibson, 1968 see footnote 5). The use of the idea of “information” brings certain problems^6^, but at this point we are tracking the ecological critique of the idea of a retinal image as it was presented at various times in work by J.J. and E. J. Gibson.

The ecological approach to perception assumes that the inputs of sensory nerves are incidental to perception because perceiving takes place by the action of perceptual systems that function as the whole organism acts in its surround (Gibson and Gibson, 1972). By 1979 Gibson had articulated the fallacy of the image in the eye:

“Ever since someone peeled off the back of the excised eye of a slaughtered ox and, holding it up in front of a scene, observed a tiny, colored, inverted image of the scene on the transparent retina, we have been tempted to draw a false conclusion. We think of the image as something to be seen, a picture on a screen. You can see it if you take out the ox's eye, so why shouldn't the ox see it? The fallacy ought to be evident…The question of how we can see the world as upright when the retinal image is inverted arises because of this false conclusion. All the experiments on this famous question have come to nothing. The retinal image is not anything that can be seen. The famous experiment of Stratton (1897) on reinverting the retinal image gave unintelligible results because it was misconceived” (Gibson, 1979, p. 62).

The idea that the retinal image is the basis of vision is only a hypothesis. There are not just logical problems with the idea, but empirical ones. Organisms without chambered eyes nevertheless do see (e.g., insects). We would add that the idea of a retinal image is only possible given the Cartesian/Newtonian accounts of light and optics.

In contrast, Ecological optics focuses on the level of the whole organism living and acting in its natural surround, and the structured arrays (in light, sound, pressure) that are consequences of the structure of the surround. These arrays are what perceptual systems are sensitive too, which allow the organism to detect, coordinate with, and change its surround as the organism goes through its life over time. Gibson, in the case of vision, describes light-filled spaces, that is, ambient arrays, structured by the surfaces, layouts, objects, and events in the organism's surround, including other organisms. The ambient structured stimulation available in the sea of energy around us is quite different from signs or signals. “The arrays to which perceptual systems resonate is not transmitted, does not consist of signals, and does not entail a sender and a receiver. The environment does not communicate with the observers who inhabit it. The world is specified in the structure of the light that reaches us, but it is entirely up to us to perceive it (Gibson, 1979, p. 63).

**Figure 3:**
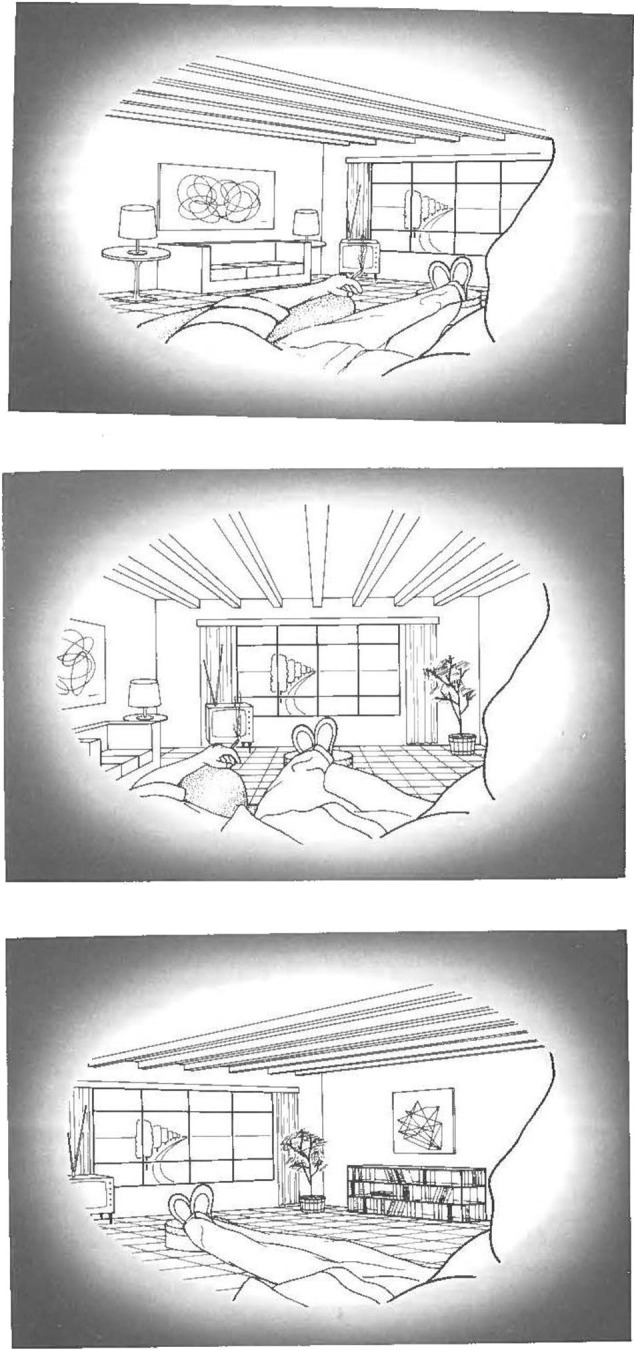
Segments of the view from the left eye as the head is turned (From Gibson, 1979, reprinted with permission).

An example of the ecological account of perceiving over time and the structure of arrays is given below in a sequence of drawings (from Gibson, 1979) evoking the perceptual experience of seeing a room as one turns one's head (see Figure 3 below, first, second, and third panels), which is an example of visual kinesthesis. The nose is always in view, and the field of view is a “sliding sample” of the ambient array with texture accreted and deleted at the leading edges. The contrast with the stationary views presented above, and especially with the inferences drawn by Mach, is clear^7^.

Ecological optics has offered a relational approach to the foundational conditions of understanding vision. This work was essential in rejecting representationism and dualism (cf. Shaw, 2001). Ulric Neisser concluded that Gibson's ecological optics was “a revolutionary step that rendered geometric optics obsolete. The idea that sensations are the building blocks from which a meaningful world is constructed is replaced by the notion that visual proprioception, or ego motion, and invariants in the optical array are central.” (Neisser, 1977 p. 253). In current day contexts, we add that ecological optics replaces the idea that sensation and motor activity are somehow linked and then form the basis of the organism's interaction with the world.

### Perception in Single-Celled Organisms: Applying the Ecological Framework

The details of perceptual systems that resonate to structured arrays has been elaborated in work that followed on Gibson's ecological theory. Analogies were made to instruments that respond to higher order variables without calculation of lower order variables, for example, the polar planimeter that measures area directly, i.e., without calculating length and width (Runeson, 1977). But an example from a living organism will be described here, as it contrasts directly to enactivist accounts given above, specifically on perception in single-celled organisms.

Bacterial behavior in relation to the chemical environment is an example of perceiving, and mechanisms or processes in this single-cell case may help to elucidate general principles of perception that pertain to all perceiving organisms (Pittenger and Dent, 1988). The basic ecological idea is that organisms attune to arrays structured by the environment in order to keep contact with the layout of the environment and to act adaptively in and on the layout of the surround. Action, in turn, provides stimulation that furthers the organism's contact with the environment. Free-living *e. coli* bacteria have basically one possible way to adapt to changes in the chemical environment in which they exist, and that is movement. Movement takes place by rotation of flagella distributed around the cell and the flagella alternate between coordinated rotation which leads to the formation of a bundle and movement in a straight line, and uncoordinated rotation which leads to the cell tumbling randomly. When these two types of movement alternate the cell moves in a zig zag fashion, but without control of the direction of any particular line segment. Decreases in the probability of a tumble increase movement in one direction, that is, toward a favorable chemical gradient; increases in the probability of a tumble decrease movement in any particular direction and, therefore, away from repellant gradients. The only way the organism can optimize its position in the environment is by relating to a *gradient* of concentration, not a local concentration, and a gradient exists over time, that is, consists of change over time in relation to the single cell. This is because the cell is so small that differences in gradient over the length of the cell, that is, a spatial gradient, are negligible. One model of the control of flagellar rotation (Koshland, 1977) posits a regulator substance within the cell that is continuously forming and decomposing, but at slightly different rates. The change in these rates results in increase or decrease in tumbling, which results in the cell probabilistically moving up or down a temporal gradient of concentration of a particular chemical in the surround.

There exist several parallels to perception in more complex organisms that make the bacterial case important in theorizing about mechanisms or processes for the direct perception of change over time as an organism coordinates with its (changing) environment. Stimulation from certain aspects of the environment is available only when the organism is moving, in vertebrates examples include accretion and deletion of texture in vision, head movement in auditory localization, and active touching in haptic exploration.

These parallels aptly illustrate the general important point that perception is of change, *per se*, and requires action and active exploration on the part of the organism. Finally, even in bacteria experience affects perception and action, i.e., certain receptors are universal, but others only develop on the cell when certain compounds are in the surround (Koshland, 1979). A direct theory of perception would describe these examples as representing the process of “resonance” to relations in the environment, as opposed to enactivist theories that describe the chemical processes that “create” the distinct unit of the cell.

### Interim Conclusions

How does the example of chemotaxis in bacteria as the direct perception of change relate to the enactivist description of bacteria as a living system? Both emphasize biological functioning as active and ongoing, as adaptive to the surround, and as unmediated, that is, as functioning without hypothetical mediational processes such as copying, storing and comparing. But the two approaches diverge in that the living systems approach does not distinguish *levels* of life inasmuch as the processes described apply at all levels from cellular to societal.

There are, however, those who differentiate the level of the organism from other levels (e.g., Denton et al., 2013), and further, those who differentiate perceiving/acting organisms from other living organisms (e.g., Gibson, 1966, 1979). The genetics and cell biology of cells within organisms has been a major focus of research, but we still know little of the ways that the activities of individual cells are orchestrated and coordinated in order to lead to not only large-scale patterns, but to actual morphology of organisms (cf. Levin, 2012a). In other words, organisms do not reduce to the functioning of individual cells that make up the organism. One consequence of this idea is that it is organisms, not their constitutive cells, that perceive and act, even if the organism consists of only a single cell, and even if it is a prokaryotic cell (e.g., *E. coli*.).

If perception is defined as a sensorimotor process, then it is carried out by *cells*, at least at some level (i.e., the retinal cells). But if perception is defined as direct resonance to relational stimulation structured by the surround of the organism, then it is carried out by the *organism* with the appropriate perceptual systems, where perceptual systems include sensitive tissues and whole bodies. These two approaches to perception have very distinct consequences for theorizing about what abilities an organism might possess that “build on” perception.

On the Varela living systems approach, living is cognizing and action is cognizing, and the “sensori” is always directly connected to the “motor” as a basis for any action. On the direct perception ecological approach perceiving is always occurring, always open and developing/differentiating/integrating, and always a direct “knowing” of the surround through acting in/on it. Direct perception leaves open the relation of perceiving and other types of knowing, and these topics are active areas of research in current ecological psychology research (e.g., McCabe et al., 1986; Dent-Read, 1997; Rader and Vaughn, 2000; Szokolszky, 2006, 2019; Araujo and Davids, 2009; Rader and Zukow-Goldring, 2012, 2015; Read and Szokolszky, 2016; Szokolszky et al., 2019). The living systems approach, in contrast, begins by defining cognition, and the definition of perception follows from that initial definition/assumption. In this case, perception is entailed, whereas on the ecological approach it is primary. Because of this difference, the ecological and enactivist approaches arrive at very different definitions of cognition (see below in Conclusions).

## Second Main Point: the Ecological Concept of the Mutuality of Organism and Environment Is Different From Sensorimotor Coupling

So far we have covered the ecological critique of and alternative to sensation-based theories of knowing/acting. In this section, we turn to how ecological and enactivist approaches understand organism-environment co-dependency. Ecological psychologists embrace “organism–environment mutuality” and enactivists refer to “structural coupling.” Although both terms imply organism–environment co-dependency, they do so, however, with very different emphases and underlying assumptions.

For ecological psychologists mutuality of organism and environment is the key to explain perception, action and cognition without recourse to representations. Mutuality ensures a deep ontological and epistemological compatibility between the organism and the environment which makes meaning inherent in the dynamic process unfolding in this relationship. Mutuality works at the level of the organism, therefore, this level is of distinct importance in the ecological explanation. Ecological Psychology has developed elaborate mutualist concepts. Gibson was at pains to show that perceiving/acting organisms and their surround are not separate, and, therefore, do not have to be “coupled” or “conjoined,” especially not by some kind of code or mental representation. “… the terms “affordance” and “ambient optic array” bridge the gap between animal and environment, because they point both ways “(Gibson, 1982, p. 234). Gibson essentially says there is no *relation* of organism and environment; he has defined it out of existence. To support this idea we reintroduce Dewey's phrase “unity of function” (see Costall, 2004, p. 191). Dewey states:”. it is quite necessary to start from the unity of function and see that the distinction of organism and environment arises because of adaptation in that process, not vice versa” (Dewey, 1976, p. 275, quoted in Costall, 2004). In life, organism and environment are not in relation; in analyzing the whole, we *form* the two parts out of our observations.

Ecological psychologists worry that enactivists do not appreciate the depth of the ecological mutuality principle and, therefore, they introduce concepts like “sense making” that might take us back to the dualism of subjective vs. objective. Fultot et al. (2016) point out that enactivism offers in fact a “physicochemical” level of description (at the level of sensations and brain activity, the subpersonal level) and a subjective level of description which implies internalism. On the other hand, the enactivists are looking for “something more than what can be provided by an ecological psychology framework on its own” (Stapleton, 2016, par.5). They question whether instead of “naturalizing subjectivity” Ecological Psychology rejects it all together, along with such organismic functions as autonomy and active contribution on the part of the organism. Ecological Psychology uses the verb “to act” while Enactivism uses “to *en*act” to characterize meaningful action. “To act” in the ecological sense implies activity of the organism. The use of “enact” is intended to imply that the act brings forth (generates, produces) meaning. Given their priority of enactment and sense making, enactivists' main critique is that the ecological concept of mutuality does not accommodate this important aspect. They also think that Enactivism provides a deeper explanation not just because of its richer sense of agency, but also because Enactivism addresses sensorimotor coupling at a variety of levels, including cellular–microenvironment, organism–environment and organism–organism levels (Stapleton, 2016).

We propose that regarding the above questions there are true differences between Ecological Psychology and Enactivism, there are apparent differences, and there are issues in both frameworks that need to be answered and elaborated. Next we lay out these aspects, elaborating the ecological concept of mutualism and also addressing the questions cited above.

### The Ecological Concept of Mutualism: The Primacy of the Animate Organism

In this section, we discuss the ecological concept of mutualism in direct contrast to the enactivist idea that to perceive is to be “in interactive relationship with the world” (Roberts, 2018). In other words, mutualism is not interaction (cf. Still and Good, 1998). What is mutualism in the Ecological sense? The term “ecological” refers to the distinct level of the animate organism as an organized whole and the axiom of mutualism: that the organism and its evolutionary and developmental environment mutually define each other. The organism (animal or human) and its environment are codefining and inherently interrelated with each other; environments are animal referential, and organisms are environment referential. Mutuality is defined at the ontological level as codependence, coregulation, codetermination, and coevolution of the organism–environment system. Mutualism in ontology entails mutuality in epistemology. The organism–environment relationship necessarily is based on reciprocity, agency, and functional significance (see, e.g., Alley, 1985; Heft, 2013; Read and Szokolszky, 2018; Szokolszky and Read, 2018).

Gibson worked out his ecological approach to perception based on the idea that the environment to be perceived should be analyzed at the level of the (animal or human) organism (e.g., 1966, 1979). On this view perceiving is an ongoing process of resonating to energy that is directly structured by the layout and surfaces of the environment (which includes other acting organisms), and, therefore, directly perceivable as the organism goes about its activities, some of which change the surround. Perceiving and acting are continually mutual.

The organismic level of analysis in biology was common before the 1950's when concepts and metaphors from engineering, specifically communication theory, began to affect biology and psychology (cf. Kay, 2000; Reisch, 2005; Keller, 2010). A few influential biologists have kept arguing, however, for the importance of the organism as a whole (e.g., Waddington, 1942, 1957; Goodwin, 1982; Webster and Goodwin, 1996; Lewontin, 2002). Ecological Psychology complements these biological theories because it studies not just animate organisms and their mutual surround, but the unity of the organism and the environment, i.e., direct perception^8^. Direct perception is possible and necessary because no animal could exist without an environment surrounding it, and, equally, an environment implies an organism (Gibson, 1979; Costall, 2004, 2011). Psychology pertains to animate organisms, so in our field, an environment implies an animate, perceiving/acting organism.

Perceiving is “adaptive” in two senses: (1) through perceiving the organism adapts to the layout of surfaces and to events by resonating to structure in the ambient energy as it moves and acts and reacts, and (2) because perception is direct, it allows the organism to live its life—to stay alive, to develop, and to provide the functions it does for its ecological niche/community^9^.

The adaptivity of the organism is by no means passive adaptation to existing circumstances. Gibson's idea of affordances and the perceiving of affordances is a mutualist account of the organism in its conditions of life. The animate organism “is a perceiver of the environment and a behavior in the environment. But this is not to say that it perceives the world of physics and behaves in the space and time of physics.” (Gibson, 1979, p. 4). Through Gibson's work, the “conditions of life” can be specified and elaborated with reference to the organism's perceptually guided action and action-supported perception.

Direct perception, by way of affordances, has begun to influence biological thinking as well, especially in relation to evolution (e.g., Walsh, 2015). Walsh (2015) characterizes proper organismic development as depending upon “the capacity of organisms to assimilate, integrate, and orchestrate the causal contributions from genes, epigenetic structures, tissues, organs, behavior, and the physical, ecological and cultural setting” (p. 157). At least at the level of behavior (action) and its ecological and cultural settings, direct perception is critical to this process. Consistent with this view, brain, mind, and consciousness are different aspects of an emergent evolutionary production, a form of life (Pickering, 2016). Adopting a niche construction perspective, Withagen and van Wermeskerken (2010) also reexamined the role of affordances in the evolutionary process. They argue that affordances and their utilization, destruction, and creation are central elements in evolutionary dynamics. These views are consistent with the idea that mutualism is a perspective on meaning that encompasses formal cause, evolutionary emergence, and ecological realism. Here Ecological Psychology aligns with the biology of Goethe and D'Arcy Thompson, both of whom studied dynamic formative processes, and are represented in modern biology by such researchers as Goodwin (1982); Levin (2012b), and Tung and Levin (2020).

### Grappling With Mutualism

Mutualism is not an easy concept to grasp. Researchers who work within Ecological Psychology, and some in related disciplines such as certain approaches to developmental/evolutionary psychology have wrestled with ideas about organisms and their surrounds (e.g., Järvilehto, 2009; Oyama, 2009; Turvey, 2009). And it is important to delineate distinctions among these approaches. Although the term “mutualism” is not always used the idea of the organism-environment system, a related concept, is central to some epigenetic, development, and ecological research. For example, Järvilehto (2009) states that:

“The environment is not just a passive scene in the background of the acting organism but an active part of the system making specific results of behavior possible. Subject and object are inseparable and represent only distinctive points of view in the organization of the system. Subject is the system in action, object is what emerges as the result of this action” (Järvilehto, 2009, p. 116).

In an independent area of research, Developmental Systems Theory (e.g., Oyama, 2009, 2012), the organism and its environment form a system with an inside and an outside which define and specify each other and codetermine outcomes. This in contrast to autopoietic systems (Maturana and Varela, 1988) which distinguish between the internal specifying power and the external triggering power of different aspects of the system. In Developmental Systems Theory there is no causally sufficient self-making but, instead, mutually constructed relations of developing organisms and their environments. We note here, that the system is taken as primary, not the organism.

Developmental Ecological Psychology shares with any developmental approach the emphasis on change over time, but it does *not* make all levels of functioning equivalent (e.g., the cell and the whole organism), and it does *not* assume sensation as the primary contact with the surround (e.g., Read and Szokolszky, 2018). Developmental work from within Ecological Psychology endeavors to place the mutuality of organism and environment into various time scales.

Mutualist concepts are themselves flexible and evolving, as the above variations attest (Still and Good, 1992, 1998; Dent-Read and Zukow-Goldring, 1997; Fultot et al., 2016; Pickering, 2016). Some points of uncertainty are important: even the idea of interrelations or interaction may require admitting the existence of two separate kinds of entities (i.e., organism and environment as entities). That is, if one *begins* with parts already analyzed, then the parts have to be related to make a system or a whole. Even Gibson (1979) used terms such as “complementary” and “reciprocal” interchangeably, but that should be clarified. “Reciprocal” indicates a relation in which one act is given in return for another; and a “complement” is something that completes, so the environment completes the organism (cf. Dent-Read, 1997).

Complementary mutualism in Ecological Psychology demands a consistent and ongoing interdependence of entities. The idea of the organism and environment as *complementary* implies a unity of function that existed before the analysis into parts. Whereas, Dewey linked the “sensory” and the “motor” aspects of organisms in continuous arcs, Gibson describes perceptual systems of the organism that exist within the organism-environment mutuality as a whole, in which the organism and environment mutually constitute each other. Still and Good (1998) list three requirements for a mutualist theory and the language used to describe it. (1) The concepts and descriptions are not primarily about either the organism or the environment, rather they relate to activities that necessarily involve both, therefore, terms are interdependent; (2) Units retain properties of the whole and (3) explanations are diachronic rather than synchronic. These aspects can serve as a basis for comparing approaches outside of Ecological Psychology, in this case, of Enactivism.

### Gibson's Phenomenological Method and the Concept of Mutualism

Gibson used phenomenological methods to investigate the perceived surround, for example, the view from one eye (see the revision of Mach's figure, Figure 3 above) to show that we always perceive our noses in any act of (unrestrained, natural) visual perception. Therefore, even when one is holding still (which is an act), and nothing else of one's body is in view, part of one's own body is always in view. If we include two eyes, we have two opposite views of the nose and, in a way, a midline is formed, even in a static view of a static scene. Even this reduced case is an example of *visual kinesthesis*^10^. Gibson went on to describe what there is to be perceived by describing the layout of the environment in organism-relevant, that is, ecological terms such as texture gradients, flow from the still point of future contact, accretion and deletion of texture at an edge, and, specifically, what was variant and invariant in these perceived structures. Optical structure “guides locomotion by specifying both the invariant surrounding surfaces and the movement of the organism within them.” (Gibson, 1966, p.163). Still and Good point out that “Visual kinesthesis retains the flow of activity; it links organism and environment dialectically, …; it applies directly to the whole organism; and, by being a part of activity necessarily extended over time it is a foundation for diachronic rather than synchronic explanation.” (Still and Good, 1992 p. 114). Along the same lines, the principle of mutualism has been described as a relational thinking encompassing non-disjunctive distinctions, for example, organism and environment (Costall, 2001). Without this type of thinking, dualisms persist, of subject and object, of agent and world and of the intentional and the material (Costall, 2001, p. 481).

The mutualism of organism and environment was the basis of Gibson's (1966) development of the idea of affordances for *animate* organisms. “Some sources (in the surround) are beneficial some noxious. If the specification is real and if the information is detected and discriminated the individual will be able to detect the values of things at a distance and move toward or away from them in accordance to what they afford.” (p. 73).

Gibson (1979) developed the concept of affordance—what the environment affords the organism in support of action, nutrition, social action, and so forth, and proposed that affordances are specified in the energy arrays that an animal's or human's perceptual systems resonate to as they move through and adapt to and change their surround. This idea clearly meets the three criteria of relational concepts, units with properties of the whole, and diachronic explanation. The term affordance “points both ways” i.e., to the environment and to the specific organism and so is interdependent; it retains properties of the whole organism-environment system inasmuch as the whole organism in its surround is involved with any particular affordance; and affordances exist over time, that is, are not snapshots and are, therefore, diachronic. The relation between invariants in the energy array over time and the affordances available to perception is a complex theoretical and empirical problem. As of now, there is no agreed upon theoretical specification of affordances that all ecological psychologists or philosophers subscribe to. Work on this problem is ongoing (e.g., Dotov et al., 2012), and, clearly, researchers endeavor to maintain mutualist thinking (with various degrees of success and consistency).

The idea of affordances has been taken in several different directions since Gibson's initial descriptions, and a review of this important topic is beyond the scope of the present paper. But we do here give brief indications of some of the diversity of approaches. The environment scaled to the organism has been investigated as an example of the range of affordances for action (e.g., Warren, 1984), the distinction between dispositional and occurrent properties of objects (Turvey, 1992) has been argued, and the question of the existence of affordances independent of any particular organism (Noble, 1981) has been raised. Later topics include the role of the organism's intentions in specifying affordances, especially social affordances (Heft, 1989), “in the course of the individual's on-going activity, particular affordances will be experienced (i.e., actualized) in conjunction with particular intentional actions; these affordances both complement and constrain these intentional processes” (p. 25). A recent enactivist paper relates affordances back to some basic Gestalt ideas (Kiverstein et al., 2019; See also, Rietveld et al., 2018) and distinguishes between the geographical environment and the behavioral environment, in other words, the environment as perceived by an individual and the shared publically available environment. The authors argue that the two environments are reciprocal and dependent. The challenge for this approach is to avoid the problems inherent in placing the geographical environment within the (human) self-consciousness, and, therefore, removing the inviting affordances from the environment (cf. Webster, 2020) and negating Gibson's initial insight. Finally, any research on affordances must avoid the fallacy of taking an outcome of a process as the pre-existing source of the process. That is, if organisms engage their surrounds in stable and predictable ways, then it is easy to assume that the qualities of the environment involved in this process are stable and have an existence previous to the engagement (van Dijk, 2019) (cf. Heider's influence on Gibson's idea of affordances, de Jong, 1995). One way to avoid this fallacy is to take seriously the idea that perceiving takes place over time, and that events occur at different time scales. A human life is the longest time scale for an individual, and everything they experience takes place on that scale. In that sense there is no “here and now” as opposed to “there and then” on which to base particular vs. general affordances (cf. Shaw et al., 2019). There is only perceiving of persistence over time, by either any individual organism, or by a trained psychologist in their scientific work.

Ecological Psychology has been critiqued for tendencies to overemphasize the environment, and even to think of the environment as preceding the organism (cf. Costall, 2004). There is, however, nothing in the Ecological approach to perception that requires this view of the environment, in fact, such a view is counter to the theory. Ecological Psychology benefits from efforts to counter the idea that the environment exists before the organism, and the organism is the one that “adapts” to a pre-existing environment. As Dewey (1898, p. 283–284, cited in Costall, 2004) points out, the environment of an organism is a product of the process of development, it has developed *along with* the organism. *Mutuality is not interactionism*, that is, the interrelating of two separate entities. Organisms inherit environments as much as they do genes, and environments exist because of organisms. The organism is different from the environment, from its surround, but this distinction “presupposes their relation, just as riverbeds and rivers, and beaten-paths and walkers imply one another's existence” (Costall, 2004, p. 191).

### Gibson's Idea of Visual Kinesthesis vs. Merleau-Ponty's Ideas of the Lived Body

In this section, we elaborate the concept of visual kinesthesis as a good case with which to illustrate mutuality in concrete terms. This concept also offers the opportunity to compare Gibson's ideas to those of Merleau-Ponty on the lived body, as the latter is often taken as foundational in Enactivism. Visual kinesthesis is perceiving the effects on what one is seeing due to one's own movement. It concerns our “awareness of being in the world” (Gibson, 1979, p. 239). For example, there are the experiences of optic flow, awareness of movement or stasis, and the visible horizon that corresponds to eye level. The visible horizon is neither objective nor subjective; it is a correspondence of distant surround and perceiver.

Still and Good (1998) discuss the ontology of mutualism by linking direct perception to Merleau-Ponty's idea of “objective co-variation” (p. 53). Merleau-Ponty describes his experience as his right hand touches his left: When my right hand touches my left, I am aware of it as a “physical thing.” But at the same moment, if I wish, an extraordinary event takes place: here is my left hand as well starting to perceive my right, …The physical thing becomes animate" (Merleau-Ponty, 1942, p. 166).

Compare this description to Gibson's characterization of visual kinesthesis: “In visual kinesthesis…the nose and the body are visible. There is information for coperceiving the self as well as for perceiving the layout” (1979, p. 84). Independent of the fact that Merleau-Ponty in this example focuses on touch (although we assume he is looking at his hands as they move and reconfigure) and Gibson is focused on vision, Merleau-Ponty takes a “first person” stance and describes his own experience, whereas Gibson takes a “third person” stance and describes vision and movement in general. Nonetheless, Gibson's description strives to be a mutualist description, by simultaneously taking into account the animate, moving organism and the layout through which it moves and which supports and, in some ways, forms its movement.

Is Merleau-Ponty's description mutualist? Taken in the context of another of his statements, viz., “I am the absolute source, my existence does not stem from my antecedents, from my physical and social environment; instead it moves out toward them and sustains them…” (Merleau-Ponty, 1945, p. ix, quoted in Still and Good, 1998, p. 54) one could argue that Merleau-Ponty is not describing a mutual relation of his self with his surround, but only his self, or at least, only his own awareness/consciousness. If Merleau-Ponty's goal in *Phenomenology of Perception* is to define and elaborate the lived body of pre-predicative experience and to distinguish it from the objective body of science (Still and Good, 1998, p. 54), then his goals and methods are completely distinct from Gibson's, which were to study how we perceive/act in the course of daily life, or in controlled conditions^11^. Based on Merleau-Ponty's early writings on *The Structure of Behavior* and *The Phenomenology of Perception* Bullington (2013) describes phenomenology as the “systematic study of the realm of subjectivity. Phenomenology does not study the objective world as such, but rather the subjective foundations for being able to experience the world as objective and independent of our acts of attending and understanding” (p. 20). This approach seems to presuppose a separation between perceiver and world, objective and subjective world, and to concentrate only on the *experience* that what is perceived is the objective world—the experience of the appearances of the world.

In the American pragmatist tradition to which Gibson was heir activity takes precedence over ideas and the goal is to develop an ontology based on activity rather than on subjective ideas and sensations. If we return to the example of the hands and touching, the ecological approach would say: when I move my right hand to touch my left, seeing my body and surround as I do this, my right hand touches actively and my left hand receives touch passively. In both cases visual kinesthesis and tactile kinesthesis are ongoing, but one acts and the other rests. The hands, eyes, head, limbs, body, and so forth are all part of the visual and tactile perceptual systems that coordinate with the layout of the surround, including one's own body in the ongoing process of direct perceiving/acting. The ambient energy arrays directly structured by the environment and one's own body are available to the animal or human perceptual systems that can attune to the arrays. Invariants specify the layout, that is, the objects, surfaces, and events in the surround and the layout consists of affordances that are enacted or not as an animal or person goes through their lives using, changing, and contributing to their surround, including other organisms.

The third-person stance of traditional science and of experimental psychology is not necessarily mutualist, but it can be mutualist when the self and the world are observed simultaneously. This is precisely the method used by Gibson in which the self-in-the-world is experienced, described, and studied through experiments. For example, in his descriptions of “size” and “distance” perception as the detection of “equal amounts of texture for equal amounts of terrain suggests that both size and distance are perceived directly” (1979, p. 162), that is, in relation to the observer. Second-person approaches, in which anything other than self is “you,” by definition, sees agency in everything in the world, living or not, animate or not, unless the work is specifically limited to human social interaction. But the living animate organism is the starting point of Ecological Psychology as an approach to perception, and so within Ecological Psychology only the mutualist “person” is fruitful. And the mutualist person can be first person, “I see,” second person, “you see,” or third person “seeing is taking place.” We should point out in summary at this point, that Gibson used the general “I see” as a method for theory development, but the third person stance in his research. The first and second person stances have yet to be further developed in Ecological Psychology, although there is an extensive literature on the second person aspects of human social interactions (Marsh et al., 2009a; Schilbach et al., 2013; Schilbach, 2015).

## Third Main Point: Ecological Psychology and Varieties of Enactivism Are Laying Down Different Paths to Pursue Related Goals

So far we have uncovered some crucial points on which Ecological explanations differ from the Enactivist explanations. Next we consider sensori-motor enaction directly, and examine its compatibility with Ecological Psychology. We go back to Varela et al. (1993, p. 202–204) criticism of Gibson because it illustrates points of divergence between ecological theory and enactivist, not in what is said there directly, but in the differences between how ecological theory is described there and in how it is described by Gibson.

First, Varela et al. (1993) refer to the sensorimotor capacities of the animal, whereas Gibson as of the 1966 book (The Senses Considered as Perceptual Systems) was clear that the senses and perception were two different levels of phenomena. Further, Varela et al. state that affordances, which they define as interaction possibilities of the world, are distinctly ecological features of the world. However, Gibson is clear that affordances are (at least) action possibilities for animals and humans, and are always taken with respect to the animate organism, that is, always “point both ways,” i.e., to the animal and the world (Gibson, 1979, p. 129), whether or not they are acted upon.

The core question is: what the two different views mean by “perceptually-guided action.” Varela et al. take perceptually-guided action as defined by Gibson as the picking up or attending to invariances in the ambient light that specify their environmental source. However, Gibson specifically refers to invariances that exist because of the animal's locomotion, grasping, looking, tool use, and so forth; and these types of activity are what is meant by “perceptually-guided action” (not the picking up of invariants) (cf. Szokolszky et al., 2019). Of course, invariants of motion in the surround also exist (see material on events above). Adaptive, purposive action can be perceptually guided because organisms have the perceptual systems to perceive their surround by means of structured energy arrays specific to the surround. In contrast, Varela et al. go on to clarify what they mean by “perceptually-guided action”—the environment is enacted and perception is sensorimotor enactment (p. 204). They endeavor to specify the sensorimotor patterns “that enable action to be perceptually guided, and so we build up the theory of perception from the structural coupling of the animal” (p. 204).

It is apparent from these contrasts that the ecological and the enactive definitions of perceiving/perception have very little overlap. Both approaches take action as central, but their definitions of what action is and how it takes place differ radically. Enactivism concentrates on “embodiment,” whereas embodiment is implied/inherent in Ecological Psychology, but there the resemblance ends. More recent literature from the enactivist point of view retains the sensorimotor basis of perception and the definition of perceptually-guided action essentially unchanged from that given by Varela et al. (e.g., Barandiaran, 2017; Degenaar and O'Regan, 2017).

A very different definition of enaction and perception is taken by other authors who use the term “enaction” to refer to a set of theories that take action and perception to be interdependent (e.g., Gangopadhyay and Kiverstein, 2009). In this approach two different points are stressed: (1) perception and action are interdependent processes, and (2) the vehicles of perception are distributed across brain, body and world (p. 64). On this view, Gibson is seen not as an opposite to enaction, but as a forerunner. Enactive theories endeavor to understand experience as it unfolds in an embodied subject situated in an environment. In the course of that effort, some theories make a distinction between the subpersonal and the personal, or between the causal neural mechanisms of perception and the contents of experience (see above). The ecological approach to perception emphasizes perception as an exploratory and purposeful activity, and, therefore, pertains to the personal level. Enactive theories also develop accounts of the personal level, but also of the physiology and, therefore, of the subpersonal level. Herein lies the rub: on the Ecological Psychology account physiology does not constitute perception, it supports it (cf. Shaw and Mace, 2005; Read and Szokolszky, 2018). To reiterate, sensation does not equal perception.

Debates on Ecological and Enactivist theory are ongoing. Fultot et al. (2016) make clear some of the distinctions between the approaches and the attempts that are being made to align them. Fultot et al. present a certain approach to Ecological Psychology which we will call the physical systems approach. They describe direct perception as Gibson originated it, but they extend it in particular way by ignoring the organismic level and instead describing “perceiving-acting systems” instead of organism-environment systems. They claim that it is in the tradition of Gibson to “seek a characterization of perceiving-acting systems that is generic for any end-directed physical system, living or non-living” (p. 1). This step puts them in line with the Varela et al. approach to autopoietic systems, which are living or not.

The systems ecological work coincides, therefore, with some of the main goals of Enactivism, at least of the Varela variety, in using physics as the basis of biology and psychology. Systems ecological psychologists still differ from Enactivists on the possibility of direct perception, that is, direct and adaptive contact with the surround. However, perception becomes the activity of systems rather that of animate organisms in both cases. Enactivism differs even from the systems approach to ecological work, though, in emphasizing “first person” experience and in differentiating the “subpersonal” from the “personal” (with many variations on the use of these ideas, cf. Gangopadhyay and Kiverstein, 2009). Heras-Escribano (2016) attempts to show that Ecological Psychology and Enactivism are complementary because Ecological Psychology accepts the Enactivist ideas on the relation between life and cognition. This even though Fultot et al. had specifically stated they were accounting for “perception-action systems” both living and non-living.

### Animate Organism vs. Cognitive Systems

Of course, Gibson's theory and research was aimed not at all of the living, but specifically at the animate living, that is, animals and humans. Systems ecological psychologists and enactivists have extended the idea of perceptually-guided action to plants (e.g., Garzon and Keijzer, 2011; Carello et al., 2012), which are obviously living, but which are not animate (growth, even adaptive growth is not the same as action; it is tropism, cf. Read and Szokolszky, 2018). This research with plants is taken as supporting enactivist ideas of the “subpersonal.” Heras-Escribano (2016) concludes that the best interaction of Ecological Psychology and Enactivism would give the explanation of the agentive or personal level of perception to Ecological Psychology with its elaboration of direct perception, and assign the explanation of the “subpersonal,” that is, processes that shape agency through neurodynamics (for those organisms that have nervous systems) to Enactivism.

The distinction between the “subpersonal” and the “personal” seems to devolve into the distinction between physiology and experience, which might lead to the distinction between body and mind. Direct perception as developed by Gibson, that is, at the level of the animal living in its surround over time, is “between” physiology and experience. Direct perception arises out of the organism and its surround; it is a direct resonance. It is the core process that makes the organism and its surround a system at the level of the living-acting organism. Perception is not “of” anything except the changes in layout as events happen or the organism moves and acts; and the surround changes directly with the organism's activity. What is happening in the organism's physiology as it is living supports perception, but does not constitute perception. In this sense, physiology cannot “shape agency” and the agent/organism cannot emerge from the physiology. Many species must move in relation to solid objects in their surround, but they accomplish this general perceiving/acting in very different ways at the level of physiology (Johnston, 2003). For example, a human child reaching for a ball and a flying bat catching an insect are perceiving the relation of their body to an object and coordinating with the object, but the underlying physiology in entirely different in these cases. The organism's action, its “ecologically effective solution” is independent of physiology. The animal-environment system is the level appropriate for the investigation of adaptive action.

### Toward Ecological Neuroscience

If the nervous system does not cause perception, then what is its role? Ecological Psychology approaches to neuroscience are in their nascency. One approach aims to explain the perception of affordances by conceptualizing neural regions in the brain as “dispositional parts of perception and action systems that temporarily assemble” to allow animals and humans to perceive, and possibly use, affordances in the environment (Schilbach et al., 2013; de Wit et al., 2017). The problem of the relation of physiology to perception is a thorny one. From early on in his work, Gibson distinguished sensation from perception, that is, perceiving is not the having of sensations, it is the detection of the surround in the course of ongoing action by means of structured ambient energy arrays. It is unfortunate that he often called these energy arrays “information” (e.g., 1967, 1979) because that concept imports ideas about signs and communication that are anathema to direct perception theory (We will not lay out this argument in detail here, but we will also not use the word “information” in relation to direct perception. See footnote 5.) Incorporating the idea of dispositions into a theory of how neurophysiology enables of perception is one possible direction in relating neural functioning to perceiving, but dispositions belong either to the surround (as in one definition of affordances, e.g., Turvey, 1992) or, as in this case, to the perceiving organism (specifically to its brain and nervous system). In other words, dispositions entail “readiness to be” of some aspect of either the organism or the environment, but not of the resonance between them, which resonance is the defining quality of direct perception (cf. Walsh, 2015). Further, if the physiology of senses and brain “allow” perception, how is this different from defining perception as constituted of sensations? The main point here is that “allowing” perception is more than “supporting” perception. Physiology is necessary, but not sufficient, for perceiving/acting, but not any *more* necessary than any environmental aspect. Level ground, for example, allows walking, but one would not claim that it allows perception. It is the occasion for perceiving the affordance of locomotion for some animals and humans (and Daleks, who could take over the universe if everything were a flat surface).

Approaches to neuroscience from within Ecological Psychology vary considerably (see de Wit and Withagen, 2019), but there are some main themes that differentiate Ecological work based on direct perception from other approaches that take psychological phenomena to reduce to the physiological (even if the reduction is based on “emergence” out of cyclic processes). van Dijk and Myin (2019) point out that one cannot logically use the pre-existence of affordances to explain the process of perceiving affordances. If action “brings about” an affordance, the existence of the affordance cannot explain the action. Likewise, we cannot logically reify an organism's act of resonating as a nervous system resonating to ambient structure. That is, the organism's resonating does not reduce to the nervous system resonating; but it does *allow*, in the case of humans who have special skills, reflection on and understanding of the nervous system. Van Dijk and Myin use the example of evoked potentials studied during color naming in two language groups (Van Dijk and Myin, p. 262) which showed a difference in potentials depending on whether the speakers had terms that distinguished light and dark blues. First, the scientific methods used here are a refinement of resonance (attunement and anticipation), and depend on direct perception. Second, the brain is not causal in the use of color names, but coordinates with such use.

A study of infants responding to a simulated looming object (van der Weel et al., 2019) that measured visual evoked potentials and the orientation of electrical source flow showed connectivity patterns emerging and changing directions between trials. The same variable that can be used to describe the flow pattern of a looming object (tau, the ratio between image size and its rate of change, Lee, 1976) can be used to measure the rate of electrical brain activity. The authors found the two tau variables to be linearly correlated and coupled with a constant. When the infant perceives looming the information does not travel “inwards,” but, instead the infant's nervous system is getting ready to provide the possibility of resonance. Evaluating the nervous system in thermodynamic terms leads to describing it in terms of “latent states of readiness for action” (Fultot et al., 2016) which corresponds to the “open-ended fractal richness of the affordance landscape in which organisms are immersed” (p. 228). These efforts to characterize resonance are important, and to the extent that they go beyond “coupling,” they offer an alternative to “sensorimotor coupling” or “sensorimotor agency” in accounting for perception/action as it is carried out by organisms who can move, but who are embodied in such highly variable ways.

### History and Experience

Enactivists emphasize that an agent always has a history; any “sensorimotor coordination” has a history. History implies both continuity and change, and, of course, processes of change over time. Such processes of change deserve close attention (cf. Raeff, 2011; Read and Szokolszky, 2018). There are levels of time to consider—the evolution of species takes place at a different time scale than the ontogeny of individuals. Perceiving, and learning by perceiving take place at a shorter time scale than ontogeny. The idea of “history” is too broad to allow for these distinctions of time scale. And processes of change can exist at any scale; processes such as differentiation, integration, metamorphosis, morphogenesis, and so forth. Which of these is meant by history? If engineers designing a machine and then modifying it after discussion is a case of “evolution” (de Pinedo, 2016) and non-living systems can perceive/act (e.g., Fultot et al., 2016), what is the place of the perceiving animal in its (self-modified) surround? What is the place of an organism perceiving over time, even over the time scale of its whole life? These core concepts of Ecological Psychology may overlap with Enactivist ideas of “history,” but basic questions remain of what or who perceives.

Enactivism has taken experience and agency as starting points, whereas Ecological Psychology has taken adaptive perceiving/acting as its starting point. On the Enactive view the embodied cognizer is an autonomous individuality based on its material ongoing self-constitution. The idea of the embodied cognizer grounds concepts of interiority and agency. Enactivism asks: what makes cognitive systems individual subjects with their own experience and perspective? (Di Paolo and De Jaegher, 2017).

The idea of a cognitive system as an individual contrasts with the original emphasis in Ecological Psychology on the ways animate organisms perceive and act in/on their surround in the process of living. Cognitive systems are a different kind of thing than an animate organism. Organisms have boundaries, and animate organisms move on their own. Plants as organisms grow and reproduce, but growth, even though it is adaptive, is not an act. Animate organisms initiate actions, even though the actions are always in a context of other organisms, other animate organisms, and the physical world in which they live and are embedded. Animate organisms are “centers of perceptions, drives, and actions” (Grene, 1974, p. 270). Stimulus-response theory, of which sensorimotor theories are a descendant, cannot explain our experience of animals as “living centers.” Referring to Adolf Portmann's descriptions of gorillas in a zoo, Grene states: “Whatever our *theories* of animal behavior or animal evolution, we must acknowledge quite simply and factually the presence here of a center in which the living being's dealings with its environment are drawn together and from which they radiate” (Grene, 1974, p. 271). On this view, plants are organized centers of growth and form, but not of perception and action^12^.

Systems are comprised of interconnected elements, but they are not necessarily living. The mathematical idea of dynamic systems (e.g., Abraham and Shaw, 1989, 1990; Abraham et al., 1992) can be applied broadly, that is, to the living and the non-living. This has the advantage of potentially providing a very general account, but the disadvantage of blurring the distinction between the living and the non-living.

As an example of the contrast between enactivist experience and ecological perception we take the case of bodily memory (e.g., Fuchs, 2017) and compare it to the idea of direct perception of persistence over time (Gibson, 1979; Warren and Shaw, 1985). On the Enactivist view, if “memory” means “the capacity of a living being to actualize its dispositions acquired in earlier learning processes” (Fuchs, 2017, p. 337), then this capacity is due to an ongoing “dynamic coupling between body and environment.” Here memory is not based on mental representation, but on lived bodily actions that are culturally formed, learned, and carried out “without thinking.” Clearly, on this definition, body memory is dynamic both in its formation through “the body's interaction with the environment” and it's flexible reactualization in later situations. Here the emphasis is on acquired skills and habits with both objects (learning to type or play a musical instrument) and other people (turn taking, conversation, ritual). On this account, personal experience is a “feeling of sameness” and a capacity to perform over time.

### Acting and Enacting

How do these two accounts, the Ecological and the Enactivist, compare? The Ecological concentrates on a perceiver/actor and the Enactivist on a body that enacts. How does action compare to enaction? In Enactivism to ACT means to put something into practice. To ACT is to do something, move, behave, function, conduct oneself. Enaction requires that something, e.g., a statement, exists before it can be put into practice, e.g., made into a law. Action requires an entity that can move, behave, adapt. Enactivists posit an auto-poietic system that functions by enacting its history, its body memory, its consciousness. Ecological Psychology, instead of looking to the perceiver to construct a meaningful world, seeks to uncover rich, lawfully structured arrays that specify the surround to an active organism.

“The world of physical reality does not consist of meaningful things. The world of ecological reality, as I have been trying to describe it, does. If what we perceived were the entities of physics and mathematics, meanings would have to be imposed on them. But if what we perceive are the entities of environmental science, their meanings can be discovered” (Gibson, 1979, p. 33).

The richness of information (i.e., the structured ambient array) is not to be found in animal-neutral physical and mathematical variables, but in variables that concern how an animal makes its way in the world, animal-referential variables, the variables of ecological realism that are perceived not constructed. Therefore, these action-referential variables are no less objective than the physical variables based on the standard physics of mechanics or dynamics with their mathematical measurement and formalism (Gibson, 1979; Michaels and Carello, 1981) (To date the variables often used in Ecological research are described using animal-neutral mathematics based on mechanics or dynamics in physics. Animal-referential variables have received less attention. Note that time-to-contact as measured by ratios of texture accretion and deletion is, as a measure, animal-neutral. Animate organisms do not perceive physical time; they perceive the flow of events).

On the Ecological account, there is no prior something that has to be enacted, instead, there is ongoing unity of functioning of the organism-environment. On the Enactivist account the self-creating system is constantly active and creative of its form of life, its way of being in the world, its knowing. These two approaches have some similarities in their goals. For example, Enactivist approaches endorse the idea that activity is central to motivating, constraining, and characterizing perception. This has been a core theme of James Gibson's ecological approach since at least the 1950s. However, these two approaches actually “run parallel” to each other because they start from mutually exclusive assumptions, definitions, and theories.

## Conclusions and Future Directions

Do Ecological Psychology and Enactivism converge on the idea of cognition without representation? At first sight: Yes. However, given that the two begin from contradictory assumptions, and proceed in different ways and directions, our answer to this question is: No. The appearance of convergence stems from the word “cognition,” but the two approaches arrive at completely different definitions of the term. In Ecological Psychology direct perception and action is the basic way of knowing, and all other ways originate there. In Enactivism cognition is emergent out of sensorimotor coupling.

We noted that both approaches propose that adaptive behavior emerges from dynamic interactions. However, whereas Ecological Psychology has emphasized “generic agency” and lawful constraints in co-dependence, enactivists have emphasized individual contribution to meaning that is “brought forth” (enacted) by active agency, in lived experience. Ecological psychologists share with enactivists the focus on organismic activity itself as the overarching purpose of cognition, and it is, therefore, the central material of experimentation. But they do not make the extra claim that activity is the reason that organisms have to construct meanings.

The central insight of the enactivist approach is that mind is a living process (Thompson, 2007). That is, mental activity is self-producing, in the sense that the organism produces and maintains a boundary between itself and the world; it is asymmetrical in the sense that the organism does something to its surroundings across the boundary that it has itself established; and it is normative in the sense that the animal acts in accordance with norms that are established, for example, by the biological need to act in an adaptive manner (Di Paolo et al., 2017). Related is the idea that living systems construct themselves by generating the very boundary conditions that are necessary for the maintenance of their self-organization (Witherington, 2011).

Ecological and Enactivist thinkers diverge primarily with respect to the emphasis placed on the contributions of the organism to perception-action. Enactivists claim that a fundamental asymmetry in the organism-environment relationship should be credited for the existence of meaning in the world. Ecological Psychologists counter that theory must take into account both the asymmetry and symmetry of organism and environment, as well as with the role of specificational arrays that allow their unity of functioning.

With regard to an ecological approach to knowing we refer back to Gibson (1979). The theory of direct perception

“...closes the gap between perception and knowledge. The extracting and abstracting of invariants are what happens in both perceiving and knowing. To perceive the environment and to conceive it are different in degree but not in kind. One is continuous with the other. Our reasons for supposing that seeing something is quite unlike knowing something come from the old doctrine that seeing is having temporary sensations one after another at the passing moment of present time, whereas knowing is having permanent concepts stored in memory. It should now be clear that perceptual seeing is an awareness of persisting structure” (Gibson, 1979, p. 258).

The Ecological knower is an adaptive actor and explorer in the course of its life. For Gibson, perception is not a process of passive reception of information that is built up into a representation of a meaningful environment, but direct sensitivity—often made possible by exploratory activity—to an environment that is action-relevant. In brief, there are no intermediaries between the knower and the known, and what is known is at the ecological scale of the behaving organism. Specifically, ecological psychology has also taken on the problem of memory, in contrast to a cognitivism that posited mental representation (Gibson, 1979; Wilcox and Katz, 1981b) and, most importantly, the idea of perceiving over time (Warren and Shaw, 1985; McCabe et al., 1986; Read and Szokolszky, 2018). If perceiving takes place over time, then it is events that are perceived and participated in by perceivers, and events take place over very different time scales (cf. Warren and Shaw, 1985). Therefore, direct perceiving/acting takes place over very different time scales (cf. Wilcox and Katz, 1981a,b; Read and Szokolszky, 2018). Examples of perceptible events comprise motion events, such as kicking in water, and structural events, such as the development of the organism (see McCabe, 1986a,b). Clearly events such as kicking and those of development exist over different scales of time, but both are perceptible (see Gibson, 1997; Gibson and Pick, 2000). These ideas have yet to be truly mined within Ecological Psychology. Efforts to understand coordination between a person's action and various descriptions of variables in perceptual arrays have proceeded apace and coordination between perceivers has also received a fair amount of research attention (e.g., Turvey, 1990; Richardson et al., 2008; Marsh et al., 2009b; Schmidt et al., 2011). But research on how perceiving is related to knowing in a broader sense, and to visualizing, remembering, and talking or conversing is still in its infancy (e.g., Dent, 1990; Dent-Read, 1997; Szokolszky, 2006, 2019; Costall, 2010; Rader and Zukow-Goldring, 2012, 2015; Read and Szokolszky, 2016).

Although this Ecological work makes assumptions about what organisms experience, it does not explicitly bring the dimension of first person experience into its theory. Organisms act as if their experience were x, and so the act is what is important to the scientist observer.

Enactivist accounts of cognition draw on the idea that a human being or animal has to make sense of its environment. To “make sense” is to relate, to complete, to coordinate one thing with another so that “sense” or understanding arises. The enactive knower is active in making sense of its environment as it creates its life.

Understanding the distinctions between Ecological Psychology and Enactivism has the potential to clarify and, therefore, strengthen each approach in its own work. Ecological Psychology are not foes but rather, friends with distinct background and ideas, who take an interest in each other (cf. Zahidi and van Eemeren, 2016). In this sense the question of possible coordination and convergences is an important one. Our answer after pursuing the question is that convergence is not possible, but mutual clarification is a worthwhile endeavor.

## Author Contributions

All authors listed have made a substantial, direct and intellectual contribution to the work, and approved it for publication.

## Conflict of Interest

The authors declare that the research was conducted in the absence of any commercial or financial relationships that could be construed as a potential conflict of interest.

## Footnotes

^2^Interviews with Eleanor Gibson (Szokolszky, 2003), with Ulric Neisser (Szokolszky, 2013); further unpublished interviews by Agnes Szokolszky conducted in 1997, with Rober E. Shaw, Michael Turvey, Nancy Rader, Claudia Carello, William M. Mace, Reuben Baron, David Lee, Alan Costall, Sverker Runeson, Claes von Hofsten, and Gunnar Jansson.

^3^For example, the decisive test of reality is whether one can discover new details by scrutiny (1979, p. 257). See Kadar and Effken (1994, 2006), and Glotzbach and Heft (1982), for accounts of some aspects of phenomenology in relation to Ecological Psychology.

^4^It is Sensorimotor Enactivism (Ward et al., 2017) in the first place that elaborates these points in the most detail. However, sensorimotor explanations are endorsed by other enactivist theories as well.

^5^“In the modern use of the retinal image theory the connections of the neurological image, or the responses made to the units firing, determine the conscious perception, not the image itself.” (Gibson, 1968, The Puzzle of the Retinal Image, available at www.trincoll.edu/depts/ecopsyc/perils). See also, Gibson (1972).

^6^The term “information” introduces a complex conceptual tangle that we prefer to avoid. Gibson used the term in a distinct way, which was so different from other uses that it invites confusion. If perception is ongoing over time, and consists of resonance to invariants in structured arrays, then it cannot be based on either bits of code that reduce uncertainty (Shannon), or messages from the stimulus that are somehow processed and decoded to reveal the world (information processing cognition). Information in the direct perception sense also cannot be “sensorimotor” activation, as argued herein. Resonance is an active process, a single dynamic process of seeking invariants and performing goals (cf. Kadar and Effken, 2006, p. 357). Note that Oyama (2012) sees “information” in biology as a metaphor for whatever “controls” or guides the development of form in living organisms (cf. Baggs and Chemero, 2018). Withagen and van der Kamp (2010) use her work to expound the idea that “perceptual information” is not a patterned ambient array separated from a perceiving organism. Also note that the idea of an array “from a point of observation” (Gibson, 1979) is a mutualist idea (see section on mutualism herein).

^7^Note also that Gibson's illustration is more accurate than Mach's, that is, is truer to observation—the edge of the view fades gradually, it is not a sharp line. This edge is the orbit of the eye occluding the layout. Gibson's illustration would be more accurate if the edge of the nose were also gradual, and not a sharp line.

^8^But note that these biologists assume indirect perception and that sensation equals perception. Their theories would be more coherent and complete if they, instead, made use of the theory of direct perception. See also (Grene, 1995).

^9^Gibson's work on perception relates to the more ecological side of Darwin's work, in which the “conditions of life” are of primary importance (Grene, 1995, p. 141). “…two great classes of facts make me think that all variability is due to changes in the conditions of life. (1) That there is more variability and more monstrosities (and these graduate into each other) under unnatural domestic conditions, than under nature. And secondly that changed conditions affect in an especial manner the reproductive organs, those organs which are to produce a new being” (Darwin, 1836–1844, quoted in Winther, 2000 p. 425). Another key idea from Darwin is that of adaptation, that is, the possibility of the members of a species to adjust to and benefit from aspects of their environment. Costall (2001) points out that his idea undermined the dualism of subject and object that is the starting point of Cartesian mechanistic science (p. 475) and he terms Darwin's studies of earth worms an early mutualist example of “ecological” psychology (p. 478).

^10^Gibson was trained in graduate school at Princeton University by Herbert Langfeld who had been a student of Carl Stumpf's in Berlin. Stumpf originated an experimental psychology of tone and musical awareness and termed it “phenomenology.” Gibson, therefore, had training in becoming aware of one's own actions/consciousness in relation to perceiving the world.

^11^Another analysis of the “lived body” of Merleau-Ponty in relation to the idea of affordance from Gibson as two complementary sides of one process is presented in Glotzbach and Heft (1982). The body is experienced in relation to its situation and the world is perceived in relation to one's body. Our analysis highlights differences and possible contradictions between “pre-predicative experience of the body” and direct perception of one's own surround. A full characterization of the relation between Merleau-Ponty's and Gibson's work on perception is for the future.

^12^“First, Portmann is by no means alleging that “consciousness” or “mind” is to predicated of all animals, let alone plants as well. Consciousness as we experience it is one expression, one style, of centricity. Even in the human individual it forms in fact only a narrow band in the wider spectrum of mental life. And so, since even our own awareness is by no means wholly focal, we need no great imaginative effort to extend a generalized concept of sentience of some sort at least to other animals. Second, consciousness in us, or sentience in a broader sense in animals generally, is again but the inner expression of centricity as such: of the fact that organisms are centers of metabolism and development, of ordered reaching out toward an environment and taking in from it, of birth and death. It is this centered dynamic, dependent as it is on the existence of *individuals*, that *is* characteristic of all life and is not characteristic of inorganic phenomena” (Grene, 1974, p.274).
